# Oseltamivir Treatment vs Supportive Care for Seasonal Influenza Requiring Hospitalization

**DOI:** 10.1001/jamanetworkopen.2025.14508

**Published:** 2025-06-10

**Authors:** Anthony D. Bai, Siddhartha Srivastava, Thuwiba Al Baluki, Fahad Razak, Amol A. Verma

**Affiliations:** 1 Division of Infectious Diseases, Department of Medicine, Queen’s University, Kingston, Ontario, Canada; 2 Division of General Internal Medicine, Department of Medicine, Queen’s University, Kingston, Ontario, Canada; 3 Department of Medicine, University of Toronto, Toronto, Ontario, Canada; 4 Li Ka Shing Knowledge Institute, St Michael’s Hospital, Unity Health Toronto, Toronto, Ontario, Canada; 5 Institute of Health Policy, Management and Evaluation, University of Toronto, Toronto, Ontario, Canada

## Abstract

**Importance:**

Current guidelines recommend oseltamivir treatment for all patients hospitalized with influenza, but this guidance is based on suboptimal evidence.

**Objective:**

To evaluate outcomes associated with oseltamivir treatment when compared with supportive care for severe seasonal influenza requiring hospitalization.

**Design, Setting, and Participants:**

This retrospective cohort study using target trial emulation included adult patients admitted to hospital with influenza from 30 hospitals in Ontario, Canada, from January 2015 to June 2023. Data were analyzed from November 2024 to March 2025.

**Exposure:**

Oseltamivir treatment on hospital day 0 or 1 vs supportive care without oseltamivir.

**Main Outcome and Measures:**

The primary outcome was in-hospital mortality. Secondary outcomes included time to being discharged alive and readmission within 30 days. Overlap weighting of propensity scores was used to balance covariates, and a competing risk model was used to compare time to being discharged alive.

**Results:**

Of 11 073 patients (mean [SD] age, 72.6 [16.8] years; 5793 female [52.3%]), there were 7632 patients (68.9%) and 3441 patients (31.1%) in the oseltamivir and supportive care groups, respectively. In hospital, 268 patients (3.5%) and 168 patients (4.9%) in the oseltamivir and supportive care groups died, respectively, with an adjusted risk difference of −1.8% (95% CI, −2.8% to −0.9%; *P* < .001). The oseltamivir treatment group was more likely to be discharged alive (adjusted subdistribution hazard ratio, 1.20; 95% CI, 1.15 to 1.25; *P* < .001). After discharge, 645 patients (8.5%) and 336 patients (9.8%) were readmitted in the oseltamivir and supportive care groups, respectively, with an adjusted risk difference of −1.5% (95% CI, −2.8% to −0.2%; *P* = .02).

**Conclusions and Relevance:**

In this cohort study of patients hospitalized with influenza, oseltamivir treatment was associated with a lower in-hospital mortality risk, earlier discharge, and lower readmission rate, supporting evidence for the current guideline recommendation of oseltamivir treatment for severe influenza. Clinical trials are needed to definitively answer this question.

## Key Points

**Question:**

In adults with influenza requiring admission to hospital, is oseltamivir treatment within the first 2 days of admission, when compared with supportive care without oseltamivir, associated with a decreased risk of death in hospital?

**Findings:**

In this cohort study of 11 073 patients hospitalized with influenza, oseltamivir treatment was associated with an adjusted risk reduction of 1.8% for in-hospital mortality when compared with supportive care.

**Meaning:**

The findings of this study support current guidelines that recommend oseltamivir treatment for patients admitted to hospital with influenza; clinical trials should be conducted to generate better quality evidence.

## Teaser

This cohort study compares outcomes of oseltamivir vs supportive care for treatment of severe seasonal influenza requiring hospitalization among adults in Canada.

## Introduction

Seasonal influenza is a common infection that can be severe and lead to hospitalization and death.^1^ Each year, influenza causes approximately 140 000 to 810 000 hospitalizations as well as 12 000 to 61 000 deaths in the US,^2^ and 12 200 hospitalizations as well as 3500 deaths in Canada.^3^

Oseltamivir is a neuraminidase inhibitor that can be used to treat severe influenza infection requiring hospitalization.^4,5^ The US and Canadian guidelines recommend treatment of all patients hospitalized with severe influenza regardless of illness duration,^4,5^ but this guidance is based on limited evidence. To date, there is a single randomized clinical trial (RCT) comparing oseltamivir with supportive care in hospitalized patients with severe influenza.^6^ In the per-protocol population of 74 patients with confirmed influenza, there was no significant difference in clinical failures between the 2 groups.^6^ Observational studies showed a possible mortality benefit with oseltamivir in severe influenza, but most of the evidence was generated during the 2009 to 2010 influenza A H1N1 pandemic,^7^ and a newer study used a comparison group of delayed oseltamivir treatment.^8^ There is a knowledge gap in terms of effectiveness of oseltamivir compared with supportive care without oseltamivir in recent influenza seasons. Based on the suboptimal evidence, there continues to be equipoise resulting in low adherence to guideline recommendations on oseltamivir treatment for patients hospitalized with severe influenza.^9^ To add to the existing evidence, we conducted a large retrospective cohort study of patients hospitalized with severe influenza to evaluate the outcomes associated with oseltamivir treatment when compared with supportive care without oseltamivir treatment in terms of in-hospital mortality, intensive care unit (ICU) use, time to discharge, and readmission.

## Methods

This retrospective cohort study used the General Medicine Inpatient Initiative (GEMINI) database.^10^ A target trial emulation framework was used, which explicitly defines the target trial and analyzes a large observational database to emulate the target trial with the aim of estimating the causal effect of an intervention.^11^ Research ethics approval was obtained from the Unity Health Toronto research ethics board with a waiver of informed consent due to the retrospective nature of the study and use of deidentified data. We reported the study using the Strengthening the Reporting of Observational Studies in Epidemiology (STROBE) reporting guideline.^12^

### Data Source

The GEMINI database includes internal medicine and ICU patients admitted to participating hospitals in Ontario, Canada.^10,13^ Administrative and clinical data are linked at the individual patient level.^10,13^ The collected data include demographics, diagnoses, interventions, discharge, readmission, medication orders, and bloodwork results.^10,13^ Diagnoses were coded using the *International Statistical Classification of Diseases and Related Health Problems, 10th Revision, Canada* (*ICD-10-CA*) codes.^14^

### Eligibility Criteria

The study comprised patients with severe influenza, defined as influenza requiring hospitalization. We included consecutive adult patients admitted to the medical inpatient service or ICU across 30 acute care hospitals in Ontario, Canada, from January 1, 2015, to June 1, 2023. To be eligible, patients needed to have influenza as the main responsible diagnosis for hospital admission based on *ICD-10-CA* diagnosis codes of J09, J10.0, J10.1, J10.8, J11.0, J11.1, and J11.8.^15^ This algorithm of *ICD-10-CA* codes has been validated against laboratory-confirmed influenza hospitalizations using the same administrative data.^15^ Patients with bacterial or viral coinfections were included in the study.

Patients were excluded if they died on hospital day 0 or 1 because they would likely have died before a patient could have been recruited and randomized for the analogous trial. Day 0 was defined as the calendar day the patient was admitted to the hospital. Patients who were prescribed peramivir or zanamivir were excluded because both agents were alternative influenza treatments that were not relevant to this study question. Baloxavir was not marketed in Canada during the study period.

### Treatment Strategies

The intervention group consisted of patients who were prescribed oseltamivir on hospital day 0 or 1. This index frame was based on observational studies that suggested most benefit for early initiation of oseltamivir treatment on hospital day 0 or 1.^8^ The comparison group consisted of patients who received supportive care without receipt of oseltamivir on day 0 and 1.

### Follow-Up Period and Outcome

Patients were followed up starting on admission as hospital day 0 and followed up until 30 days after hospital discharge. The primary outcome was all-cause in-hospital mortality. Secondary outcomes included time to being discharged alive, transfer to the ICU after 48 hours from admission, and readmission to a participating hospital site within 30 days of discharge for any reason.

### Covariates

Covariates were baseline prognostic factors and included the following: (1) demographics (age, sex, and resident of a long-term care facility), (2) time and setting (hospital site and influenza season year [September 1 to August 31 of each year]), (3) comorbidities associated with increased risk for severe pneumonia^16^ and the Charlson comorbidity index,^17^ and (4) infection severity (influenza pneumonia, ICU admission within 48 hours from admission, and modified Laboratory-Based Acute Physiology Score [mLAPS] within 24 hours of admission).^18^ Diagnosis of influenza pneumonia was based on *ICD-10-CA* codes J10.0 and J11.0, which signified lower respiratory tract infection; this was a diagnosis made by the physician that typically required demonstration of infiltrate on chest imaging.

### Statistical Analysis

There were no missing data for the covariates used for propensity score weighting. Descriptive analysis used counts and percentages for categorical variables and mean (SD) or median (IQR) for continuous variables when appropriate. Absolute standardized difference of the mean (ASDM) was used because it is the recommended method to describe balance of baseline covariates for propensity score–based analysis and it is not influenced by very large sample sizes.^19^

The primary analysis was a modified intention-to-treat analysis, in which patients must have been prescribed 1 or more doses of oseltamivir to be included in the oseltamivir group, and patients must not have received any oseltamivir on hospital day 0 or 1 to be included in the supportive care group. If patients in the supportive care group received oseltamivir at a later time, then they were still included in the supportive care group. A per-protocol analysis was also done as a secondary analysis. The per-protocol population consisted of patients who received oseltamivir until death, discharge, or minimum of 5 days in the oseltamivir group and patients who never received oseltamivir during the entire hospital stay in the supportive care group. Both the modified intention-to-treat and per-protocol analyses had the same inclusion and exclusion criteria listed under eligibility criteria.

For the outcomes other than time to discharge alive, a risk difference with a 95% CI was calculated as risk for the oseltamivir group minus the risk for the supportive care group. A competing risk model was used to describe time to being discharge alive because it accounted for differential follow-up length and death in hospital as a competing event rather than a censoring event.^20^ Based on a cumulative incidence function, the Fine and Gray model^21^ was used to estimate the subdistribution hazard ratio (sHR) for being discharged alive, which would be associated with adjusted length of stay for those discharged alive.

Overlap weighting based on propensity scores was used to balance baseline covariates and estimate the average treatment effect for the overlap population.^22^ The overlap population is the population with a similar covariate distribution who would be eligible for the analogous trial due to clinical equipoise.^22^ The following covariates were used to estimate the propensity score using a logistic regression model: age, sex, resident of a long-term care facility, hospital site, year of influenza season, comorbidities, influenza pneumonia, and ICU admission. Overlap weighting for 2 groups always leads to an exact balance in means of included covariates and an ASDM of 0.^23^ The weighted difference in means would be the average causal adjusted risk difference (aRD) and the standard errors were used to construct the 95% CI. The overlap weights were entered into the competing risk model to estimate the adjusted sHR for being discharged alive. Bootstrap was used to construct the 95% CI for the adjusted sHR.

All 95% CIs were 2-sided. We adjusted for multiple comparisons using Bonferroni correction to set the significant threshold of *P* < .025 for the 2 sHRs and 2 secondary outcomes.^24^ Analyses were conducted using the statistical software R version 4.1.3 (R Project for Statistical Computing) and R package PSweight for overlap weighting from November 2024 to March 2025.^25^

## Results

### Patient Characteristics

**Figure 1.**
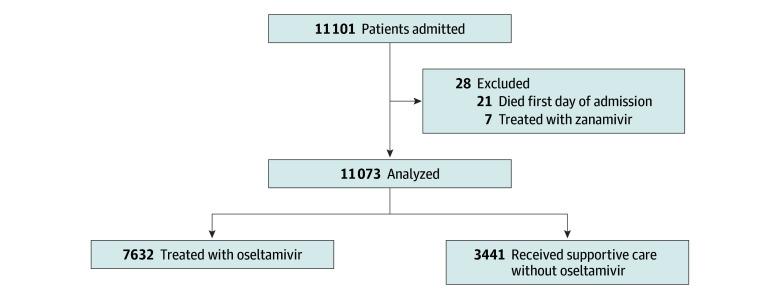
Patient Flow Diagram

Of 11 073 patients included in the modified intention-to-treat analysis (Figure 1), the mean (SD) age was 72.6 (16.8) years and 5793 (52.3%) were female. There were 7632 (68.9%) and 3441 (31.1%) patients in the oseltamivir and supportive care groups, respectively. Baseline patient characteristics are described in Table 1 and eTable 1 in Supplement 1. There were 39 patients (0.5%) and 32 patients (0.9%) with documented bacterial or viral coinfection in the oseltamivir and supportive care groups, respectively (eTable 2 in Supplement 1). The proportion of patients receiving antibiotic treatment for community-acquired pneumonia is described in eTable 3 in Supplement 1. The median (IQR) time from triage in the emergency department to hospital admission was 7.1 (5.2-9.5) hours and 7.1 (5.1-9.5) hours in the oseltamivir and supportive care groups, respectively.

**Table 1.** Baseline Patient Characteristics

Characteristic	Patients, No. (%) (N = 11 073)		ASDM
	Oseltamivir treatment (n = 7632)	Supportive care (n = 3441)	
Demographics			
Age, mean (SD), y	72.7 (16.9)	72.3 (16.7)	0.026
Sex			
Female	4004 (52.5)	1789 (52.0)	0.009
Male	3628 (47.5)	1652 (48.0)	0.009
From a long-term care facility	309 (4.1)	105 (3.1)	0.054
Influenza season			
2014-2015	84 (1.1)	92 (2.7%)	0.116
2015-2016	736 (9.6)	401 (11.7)	0.065
2016-2017	1288 (16.9)	636 (18.5)	0.042
2017-2018	2070 (27.1)	806 (23.4)	0.085
2018-2019	1359 (17.8)	722 (21.0)	0.080
2019-2020	1393 (18.3)	302 (8.8)	0.280
2020-2021^a^	≤5 (≤0.07)^b^	5-10 (0.1-0.3)	0.052
2021-2022^a^	25-30 (0.3-0.4)	30-35 (0.9-1.0)	0.080
2022-2023	674 (8.8)	441 (12.8)	0.129
Comorbidities			
Neoplasm	595 (7.8)	258 (7.5)	0.011
Liver disease	122 (1.6)	49 (1.4)	0.014
Congestive heart failure	879 (11.5)	423 (12.3)	0.024
Cerebrovascular disease	85 (1.1)	31 (0.9)	0.021
Chronic kidney disease	441 (5.8)	187 (5.4)	0.015
Chronic obstructive pulmonary disease	1979 (25.9)	1008 (29.3)	0.075
Dementia	681 (8.9)	274 (8.0)	0.035
Charlson comorbidity index, mean (SD)	1.1 (1.3)	1.1 (1.4)	0.039
Infection severity			
Influenza pneumonia	2172 (28.5)	949 (27.6)	0.020
ICU admission within 48 h	593 (7.8)	230 (6.7)	0.042
mLAPS Score within 24 h, mean (SD) [participants, No.]^c^	22.5 (17.6) [7516]	23.7 (18.2) [2735]	0.064

Abbreviations: ASDM, absolute standardized difference of the mean; ICU, intensive care unit; mLAPS, modified Laboratory-Based Acute Physiology score.

^a^ These data are expressed as ranges so that the cells with 5 or less cannot be back-calculated based on the column total or the standardized difference of the mean.

^b^ All cells that contain or reveal 5 individuals or fewer were suppressed to protect patient confidentiality as per General Medicine Inpatient Initiative data reporting policy.

^c^ Patients may not have bloodwork drawn for all the mLAPS parameters.

Hospital admissions over time during the study period are shown in the eFigure in Supplement 1. Based on national surveillance data, oseltamivir resistance was rare, ranging from 0% to 0.4% (10 of 2243 patients) during the study period in Canada (eTable 4 in Supplement 1).

Within the oseltamivir group, 5260 patients (68.9%) were taking oseltamivir on hospital day 0 and 2372 patients (31.1%) started taking oseltamivir on hospital day 1. The median (IQR) days of oseltamivir treatment was 5 (4-6) days.The overlap population based on propensity scores had an ASDM of 0 for all variables listed in Table 2 and eTable 5 in Supplement 1.

**Table 2.** Baseline Characteristics After Balancing Baseline Covariates Using Overlap Weighting of Propensity Scores

Characteristic	Patients, theoretical No. (%)^a^	
	Oseltamivir treatment (effective sample size = 6276)	Supportive care (effective sample size = 2822)
Demographics		
Age, mean (SD), y	72.4 (16.9)	72.4 (17.0)
Sex		
Female	3301 (52.6)	1484 (52.6)
Male	2975 (47.4)	1338 (47.4)
From a long-term care facility	195 (3.1)	87 (3.1)
Influenza season		
2014-2015	126 (2.0)	55 (2.0)
2015-2016	703 (11.2)	316 (11.2)
2016-2017	1111 (17.7)	499 (17.7)
2017-2018	1531 (24.4)	689 (24.4)
2018-2019	1287 (20.5)	579 (20.5)
2019-2020	715 (11.4)	322 (11.4)
2020-2021	≤5 (0.1)^b^	5-10 (0.1)
2021-2022^c^	40-45 (0.7)	15-20 (0.7)
2022-2023	753 (12.0)	339 (12.0)
Comorbidities		
Neoplasm	496 (7.9)	223 (7.9)
Liver disease	100 (1.6)	45 (1.6)
Congestive heart failure	797 (12.7)	358 (12.7)
Cerebrovascular disease	63 (1.0)	28 (1.0)
Chronic kidney disease	370 (5.9)	166 (5.9)
Chronic obstructive pulmonary disease	1770 (28.2)	796 (28.2)
Dementia	552 (8.8)	248 (8.8)
Infection severity		
Influenza pneumonia	1795 (28.6)	807 (28.6)
ICU admission within 48 h	414 (6.6)	186 (6.6)

Abbreviation: ICU, intensive care unit.

^a^ The theoretical No. was back-calculated based on the frequency after overlap weighting multiplied by the effective sample size.

^b^ All cells that contain or reveal 5 individuals or fewer were suppressed to protect patient confidentiality as per General Medicine Inpatient Initiative data reporting policy.

^c^ These data are expressed as ranges so that the cells with 5 or less cannot be back calculated based on the column total or the standardized difference of the mean.

### Outcome

**Figure 2.**
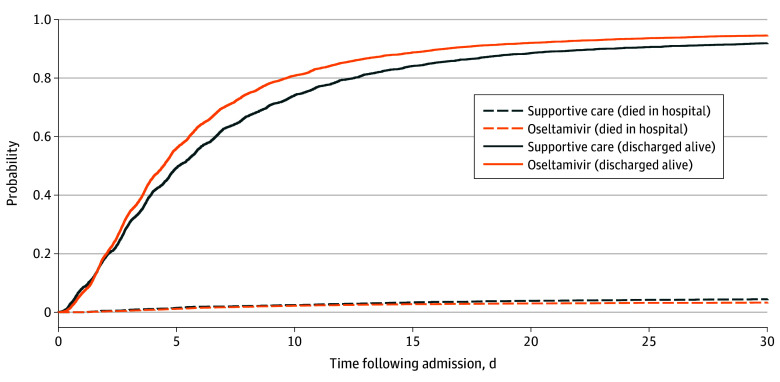
Cumulative Incidence Function for Time to Being Discharged Alive or Death in Hospital

The median (IQR) length of stay was 4.4 (2.4-7.7) days and 4.9 (2.6-9.6) days in the oseltamivir and supportive care groups, respectively. In hospital, 268 patients (3.5%) and 168 patients (4.9%) in the oseltamivir and supportive care groups died, respectively. After discharge, 645 patients (8.5%) and 336 patients (9.8%) were readmitted in the oseltamivir and supportive care groups, respectively. In a competing risk model, those receiving oseltamivir treatment were less likely to die in hospital (sHR, 0.73; 95% CI 0.60-0.88; *P* = .001) and were more likely to be discharged alive earlier (sHR, 1.18; 95% CI, 1.13-1.23; *P* < .001) (Figure 2).

The outcomes are described in Table 3 before and after overlap weighting. The oseltamivir group had a significantly lower adjusted risk for in-hospital mortality (aRD, −1.8%; 95% CI, −2.8% to −0.9%; *P* < .001), ICU transfer (aRD, −0.7%; 95% CI, −1.2% to −0.1%; *P* = .02), and readmission (aRD, −1.5%; 95% CI, −2.8% to −0.2%; *P* = .02). In a competing risk model, those receiving oseltamivir treatment were less likely to die in hospital (adjusted sHR, 0.66; 95% CI, 0.54 to 0.81; *P* < .001) and more likely to be discharged alive earlier (adjusted sHR, 1.20; 95% CI, 1.15 to 1.25; *P* < .001). The outcomes for patients admitted and not admitted to the ICU within the first 48 hours are described in eTable 6 in Supplement 1.

**Table 3.** Primary and Secondary Outcomes of Patients Who Were Treated With Oseltamivir vs Patients Who Received Supportive Care

Outcome	Patients, No. (%) (N = 11 073)		Unadjusted risk difference, % (95% CI)	*P* value	Adjusted risk difference, % (95% CI)	*P* value
	Oseltamivir treatment (n = 7632)	Supportive care (n = 3441)				
Primary						
Death in hospital	268 (3.5)	168 (4.9)	−1.4 (−2.2 to −0.6)	<.001	−1.8 (−2.8 to −0.9)	<.001
Secondary						
ICU transfer after 48 h	91 (1.2)	67 (2.0)	−0.8 (−1.3 to −0.3)	.002	−0.7 (−1.2 to −0.1)	.02
Readmission within 30 d	645 (8.5)	336 (9.8)	−1.3 (−2.5 to −0.2)	.03	−1.5 (−2.8 to −0.2)	.02

Abbreviation: ICU, intensive care unit.

### Sensitivity Analyses

Of 3441 patients in the supportive care group, 877 (25.5%) received oseltamivir treatment later from hospital day 3 to 14. Of 7632 patients in the oseltamivir group, 164 (2.2%) did not receive the oseltamivir for at least 5 days or until death or discharge. Therefore, there were 7468 and 2564 patients in the oseltamivir and supportive care groups, respectively, for the per-protocol analysis that yielded similar results (eTable 7 in Supplement 1). Sensitivity analysis based on overlap weighting of propensity scores that included mLAPS score within 24 hours (eTable 8 in Supplement 1) showed similar results as the primary analysis.

## Discussion

In this cohort study using a target trial emulation of 11 073 patients hospitalized for severe influenza, patients treated with oseltamivir were less likely to die in hospital, more likely to be discharged alive earlier, less likely to be transferred to the ICU, and less likely to be readmitted to hospital after discharge. The absolute risk reduction for mortality was −1.8% (95% CI, −2.8% to −0.9%). Therefore, oseltamivir may have a small but clinically significant benefit in severe influenza.

Our study findings are consistent with prior studies. In a network meta-analysis of RCTs for severe influenza,^26^ oseltamivir had an absolute risk reduction of −1.4% (95% CI, −2.8% to 9.7%) for mortality with very low certainty, which is very close to our study point estimate of −1.8%. In contrast, our findings were more precise and statistically significant. In a meta-analysis of 78 observational studies and 29 234 hospitalized patients with influenza during the 2009 to 2010 influenza A H1N1 pandemic,^7^ neuraminidase inhibitor treatment was associated with a mortality benefit (adjusted odds ratio, 0.81; 95% CI, 0.70 to 0.93). In contrast, our study has more contemporary data from 2014 to 2023, during which there were dominant influenza A strains other than H1N1. Newer observational studies have shown that early oseltamivir treatment is associated with lower risk of ICU admission, mortality, and readmission for severe influenza.^8,27,28,29^ However, in the largest study on hospitalized patients^8^ and ICU patients,^27^ patients in both the treatment and comparison groups received oseltamivir. Inclusion of only patients who received oseltamivir treatment likely introduced immortal time bias because patients need to survive long enough to receive a diagnosis and then initiate oseltamivir treatment. Furthermore, comparison with patients who received oseltamivir at a later time likely introduces selection bias because these patients were more likely to have deteriorated, prompting the clinician to treat with oseltamivir, and be different from patients who were never treated with oseltamivir. In contrast, our study described the benefit of oseltamivir compared with supportive care without oseltamivir treatment and accounted for immortal time bias using landmark analysis that excluded patients who died on day 0 or 1. It should be acknowledged that the landmark analysis likely does not fully account for immortal time bias. The other 2 smaller studies^28,29^ were unable to show a significant benefit for in-hospital mortality, likely due to small sample sizes.

Our study adds to the existing evidence on the benefit of oseltamivir treatment in patients hospitalized for severe influenza, which supports the current guideline recommendation of antiviral treatment for these patients.^4,5^ Ideally, efforts should be made toward RCTs to provide higher quality evidence. Given the small mortality benefit shown in our study, a trial powered on mortality would need to have a very large sample size. We look forward to the results from the ongoing Randomised Evaluation of COVID-19 Therapy (RECOVERY) trial that is comparing oseltamivir with supportive care for patients hospitalized with influenza.^30^ It will be interesting to compare the RECOVERY trial results to our target trial emulation that was designed and analyzed without knowledge of the RECOVERY trial results.

### Strengths and Limitations

Our study has several strengths. First, we had a large sample size of 11 073 patients that led to more precise estimates and allowed us to detect small but clinically important differences in mortality. To our knowledge, our study is the largest study to date comparing oseltamivir with supportive care without oseltamivir treatment for seasonal influenza requiring hospitalization. Second, the inclusion of 30 hospital sites over 9 influenza seasons make the study results more generalizable. However, the study results may be less applicable to other settings with very different patient population or practice patterns. Third, we used a target trial emulation approach that accounted for immortal time bias by excluding those who died on hospital day 0 or 1. Moreover, overlap weighting of propensity score was used to balance covariates and account for confounding by indication. Our findings were robust across several sensitivity analyses.

There are several limitations that merit mentioning. First, we did not have the microbiology data to confirm influenza. However, the algorithm based on *ICD-10-CA* codes used in our study has already been validated against laboratory-confirmed influenza using the same data source and population in a prior study with a high positive predictive value of 91% and negative predictive value of 96%.^15^ Although the diagnostic accuracy is high, it is not perfect. As a result, this study will have included some false positives and missed some false negatives, which have the potential to introduce selection bias. Similar algorithms using *ICD-10* codes have been used previously.^31^

Second, we did not account for outpatient oseltamivir treatment prior to presentation to hospital or days of symptoms as a baseline covariate. However, this aligns with the current guidelines that all patients hospitalized with influenza should be treated regardless of illness duration.^4,5^

Third, there is a possibility of residual confounding. We adjusted for many covariates including demographics, hospital sites, comorbidities, influenza season, and infection severity. It is possible that there are unmeasured confounders. One possible confounder is influenza vaccination status due to the data being unavailable. However, vaccination status data are incomplete in most studies to date.^7,8,27,28,29^ Influenza vaccination prevents hospitalization and decreases infection severity. Therefore, inclusion of only hospitalized patients and balancing infection severity covariates likely also adjusts for the effect of vaccination. Also, we could not adjust for oxygenation status because these data are not available in the database. However, in the sensitivity analysis, we estimated the propensity score based on the mLAPS score that included arterial PaO_2_ and PaCO_2_. It is reassuring that this sensitivity analysis produced similar results. We have adjusted for influenza pneumonia as an infection severity marker. We assumed that influenza pneumonia was present at baseline as the admitting main responsible diagnosis, but this could have occurred later.

Fourth, 25.5% of the supportive care group received oseltamivir later in the study, causing contamination between groups. This would make the 2 groups more similar to one another, bias the results toward the null, and make the estimates more conservative in the modified intention-to-treat analysis. It is reassuring that the per-protocol analysis showed similar results.

Fifth, our study did not capture out-of-hospital deaths. In-hospital mortality likely captured most attributable deaths. Patients who were discharged and then deteriorated and were readmitted to hospital would have been captured with the secondary outcome of hospital readmission. Our mortality rate was similar to the mortality rate from the national surveillance data (eTable 4 in Supplement 1), suggesting that we did not miss a significant number of deaths.

## Conclusions

In this cohort study, oseltamivir treatment within 2 days of admission was associated with lower mortality risk for adult patients hospitalized for severe influenza. This study adds supporting evidence for the current guidelines that recommend antiviral treatment for patients admitted to hospital with influenza while awaiting RCT results.

## Notes

Supplement 1. **eTable 1.** Additional Baseline Patient Characteristics **eTable 2.** Coinfections **eTable 3.** Antibiotic Treatment for Community-Acquired Pneumonia **eFigure.** Hospital Admission for Influenza Over Time **eTable 4.** Influenza Dominant Subtype and Influenza B Cases for Each Influenza Season Based on National Surveillance **eTable 5.** Additional Baseline Characteristics After Overlap Weighting Using Propensity Scores **eTable 6.** Subgroup Analysis Based On Initial ICU Admission **eTable 7.** Per-Protocol Analysis **eTable 8.** Outcomes After Adjustment by Overlap Weighting of Propensity Scores That Includes mLAPS Score Within 24 Hours

Supplement 2.
Data Sharing Statement
