# Supplementary material for: Oseltamivir Treatment vs Supportive Care for Seasonal Influenza Requiring Hospitalization
Source: JAMA Netw Open. 2025 Jun 10;8(6):e2514508. doi: 10.1001/jamanetworkopen.2025.14508 (PMC12152703; doi:10.1001/jamanetworkopen.2025.14508)
Supplement: Supplement 1. — eTable 1. Additional Baseline Patient Characteristics eTable 2. Coinfections eTable 3. Antibiotic Treatment for Community-Acquired Pneumonia eFigure. Hospital Admission for Influenza Over Time eTable 4. Influenza Dominant Subtype and Influenza B Cases for Each Influenza Season Based on National Surveillance eTable 5. Additional Baseline Characteristics After Overlap Weighting Using Propensity Scores eTable 6. Subgroup Analysis Based On Initial ICU Admission eTable 7. Per-Protocol Analysis eTable 8. Outcomes After Adjustment by Overlap Weighting of Propensity Scores That Includes mLAPS Score Within 24 Hours [file jamanetwopen-e2514508-s001.pdf]

Supplemental Online Content

Bai AD, Srivastava S, Al Baluki T, Razak F, Verma AA. Oseltamivir treatment vs supportive care for seasonal influenza requiring hospitalization. *JAMA Netw. Open*. 2025;8(6):e2514508. doi:10.1001/jamanetworkopen.2025.14508

eTable 1. Additional Baseline Patient Characteristics
eTable 2. Coinfections
eTable 3. Antibiotic Treatment for Community-Acquired Pneumonia
eFigure. Hospital Admission for Influenza Over Time
eTable 4. Influenza Dominant Subtype and Influenza B Cases for Each Influenza Season Based on National Surveillance
eTable 5. Additional Baseline Characteristics After Overlap Weighting Using Propensity Scores
eTable 6. Subgroup Analysis Based On Initial ICU Admission
eTable 7. Per-Protocol Analysis
eTable 8. Outcomes After Adjustment by Overlap Weighting of Propensity Scores That Includes mLAPS Score Within 24 Hours

This supplemental material has been provided by the authors to give readers additional information about their work.

© 2025 Bai AD et al. *JAMA Network Open*.

eTable 1. Additional Baseline Patient Characteristics

	Patients No. (%)		
	Oseltamivir treatment (N=7,632)	Supportive care (N=3,441)	ASDM
Hospital site			
1	304 (4.0)	67 (2.0)	0.120
2	363 (4.8)	63 (1.8)	0.164
3	≤5 (≤0.07)	≤5 (≤1.5)	0.036
4	291 (3.8)	60 (1.7)	0.126
5	472 (6.2)	118 (3.4)	0.129
6	388 (5.1)	194 (5.6)	0.025
7	494 (6.5)	128 (3.7)	0.125
8	322 (4.2)	139 (4.0)	0.009
9	218 (2.9)	57 (1.7)	0.081
10	184 (2.4)	185 (5.4)	0.154
11	340 (4.5)	111 (3.2)	0.064
12	110 (1.4)	86 (2.5)	0.076
13	322 (4.2)	111 (3.2)	0.052
14	90 (1.2)	74 (2.2)	0.076
15	298 (3.9)	217 (6.3)	0.109
16	65 (0.9)	14 (0.4)	0.056
17	121 (1.6)	85 (2.5)	0.063
18	129 (1.7)	64 (1.9)	0.013
19	112 (1.5)	57 (1.7)	0.015
20	572 (7.5)	183 (5.3)	0.089
21	562 (7.4)	138 (4.0)	0.145
22	626 (8.2)	150 (4.4)	0.159
23	38 (0.5)	107 (3.1)	0.197
24	5 to 10 (0.07 to 0.13)	349 (10.1)	0.467
25	257 (3.4)	194 (5.6)	0.110
26	7 (0.09)	170 (4.9)	0.313
27	252 (3.3)	110 (3.2)	0.006
28	363 (4.8)	121 (3.5)	0.062
29	303 (4.0)	81 (2.4)	0.092
30	15 (0.2)	5 to 10 (1.5 to 2.9)	0.008

ASDM = absolute standardized difference of the mean

© 2025 Bai AD et al. *JAMA Network Open*.

eTable 2. Coinfections

	Patients No. (%)	
Pathogen	Oseltamivir treatment (N=7,632)	Supportive care (N=3,441)
Viral co-infections		
RSV	33 (0.4)	29 (0.8)
COVID-19	≤5 (≤0.07)	≤5 (≤1.5)
Other respiratory viruses	≤5 (≤0.07)	≤5 (≤1.5)
Bacterial co-infections	≤5 (≤0.07)	≤5 (≤1.5)

Viral or bacterial co-infections were based on ICD-10-CA diagnoses codes.

© 2025 Bai AD et al. *JAMA Network Open*.

eTable 3. Antibiotic Treatment for Community-Acquired Pneumonia

	Patients No. (%)	
Antibiotic	Oseltamivir treatment (N=7,632)	Supportive care (N=3,441)
Ceftriaxone or Cefotaxime	2,917 (38.2)	820 (23.8)
Amoxicillin-Clavulanate	662 (8.7)	191 (5.6)
Levofloxacin or Moxifloxacin	976 (12.8)	280 (8.1)
Macrolide	1,784 (23.4)	503 (14.6)
Doxycycline	127 (1.7)	58 (1.7)

© 2025 Bai AD et al. *JAMA Network Open*.

eFigure. Hospital Admission for Influenza Over Time

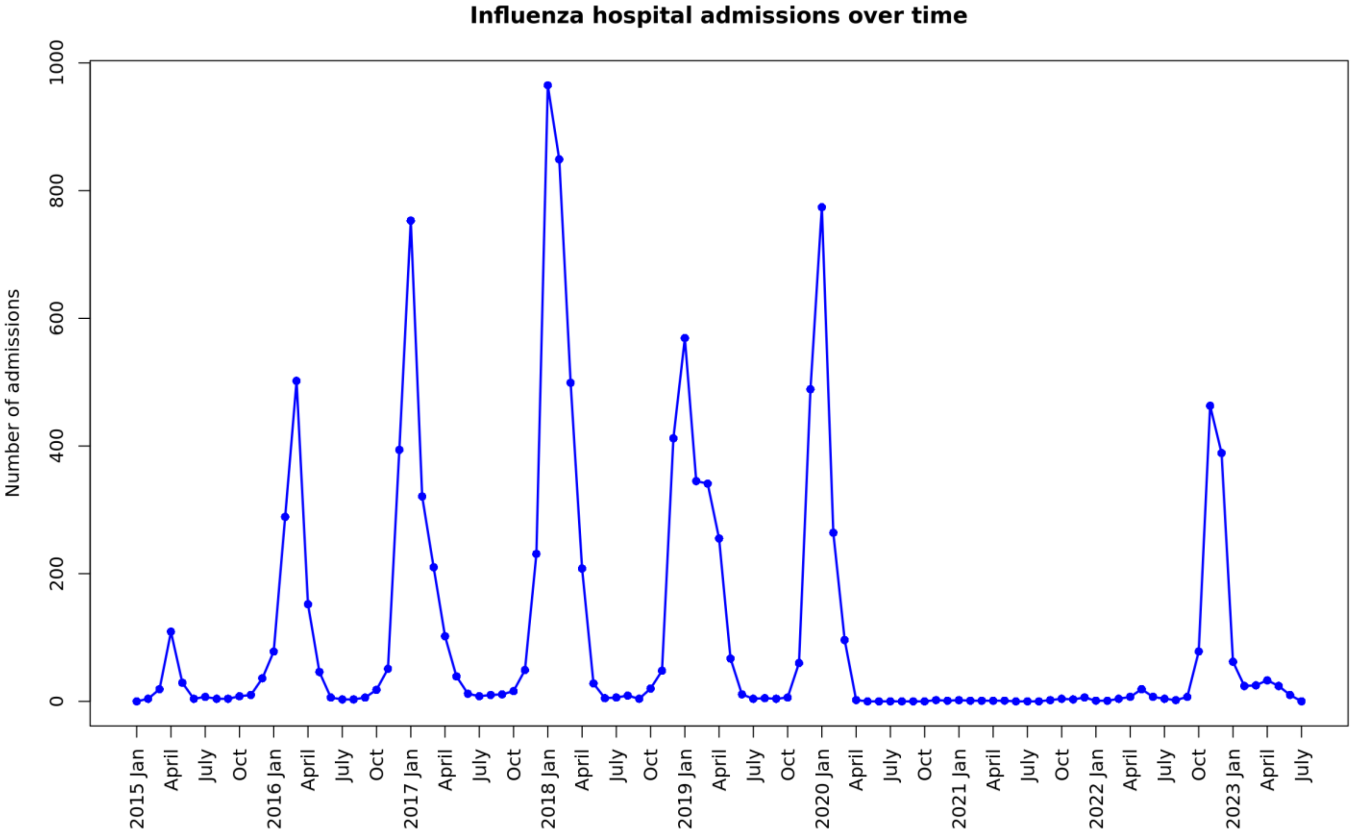

Of note, there were very few influenza cases during the 2020 to 2021 (9 cases) and 2021 to 2022 (60 cases) influenza seasons due to wide-ranging public health measures across Canada in response to the COVID-19 pandemic that led to significant decrease in influenza hospitalizations.

Reference: Groves HE, Papenburg J, Mehta K, Bettinger JA, Sadarangani M, Halperin SA, Morris SK; for members of the Canadian Immunization Monitoring Program Active (IMPACT). The effect of the COVID-19 pandemic on influenza-related hospitalization, intensive care admission and mortality in children in Canada: A population-based study. Lancet Reg Health Am. 2022 Mar;7:100132. doi: 10.1016/j.lana.2021.100132.

© 2025 Bai AD et al. *JAMA Network Open*.

eTable 4. Influenza Dominant Subtype and Influenza B Cases for Each Influenza Season Based on National Surveillance

Influenza season	Predominant strain	Proportion of Influenza B	Deaths / hospitalizations in surveillance sites	Oseltamivir resistance of those tested
2014 to 2015	A(H3N2)	19.5%	135/2,228	1/1,935
2015 to 2016	A(H1N1)	28%	55/1,153	10/2,243
2016 to 2017	A(H3N2)	11%	388/6,545	2/1,260
2017 to 2018	A(H3N2)	44%	302/5,176	3/1,767
2018 to 2019	A(H1N1)	5%	224/3,657	4/1,448
2019 to 2020	A(H1N1)	41%	120/2,493	1/733
2020 to 2021	A(H3N2)	32%	No hospitalizations or deaths	Too few to determine
2021 to 2022	A(H3N2)	1%	22/776	0/259
2022 to 2023	A(H3N2)	7%	275/4,216	0/604

Please note that the numbers above are from national surveillance data and not from the study

Reference: https://publications.gc.ca/site/eng/9.507424/publication.html

© 2025 Bai AD et al. *JAMA Network Open*.

eTable 5. Additional Baseline Characteristics After Overlap Weighting Using Propensity Scores

	Oseltamivir treatment	Supportive care
Effective sample size	6276	2822
Hospital site		
1	2.8%	2.8%
2	2.7%	2.7%
3	0%	0%
4	2.5%	2.5%
5	4.8%	4.8%
6	6.4%	6.4%
7	5.0%	5.0%
8	4.8%	4.8%
9	2.3%	2.3%
10	4.7%	4.7%
11	4.2%	4.2%
12	2.4%	2.4%
13	4.1%	4.1%
14	2.0%	2.0%
15	6.1%	6.1%
16	0.6%	0.6%
17	2.5%	2.5%
18	2.2%	2.2%
19	1.9%	1.9%
20	6.9%	6.9%
21	5.5%	5.5%
22	6.1%	6.1%
23	1.5%	1.5%
24	0.5%	0.5%
25	5.3%	5.3%
26	0.3%	0.3%
27	3.8%	3.8%
28	4.7%	4.7%
29	3.2%	3.2%
30	0.3%	0.3%

© 2025 Bai AD et al. *JAMA Network Open*.

eTable 6. Subgroup Analysis Based On Initial ICU Admission

	Patients No. (%)		
	Oseltamivir treatment	Supportive care	Unadjusted risk difference in % (95% CI)
Patients admitted to ICU within 48 hours of admission	N=593	N=230	
Death in hospital	85 (14.3)	39 (17.0)	-2.6 (-8.6 to 2.7)
Readmission within 30 days	45 (7.6)	26 (11.3)	-3.7 (-8.8 to 0.5)
Patients not admitted to ICU within 48 hours of admission	N=7,039	N=3,211	
Death in hospital	183 (2.6)	129 (4.0)	-1.4 (-2.2 to -0.7)
ICU transfer after 48 hours	91 (1.3)	67 (2.1)	-0.8 (-1.4 to -0.3)
Readmission within 30 days	600 (8.5)	310 (9.7)	-1.1 (-2.4 to 0.1)

© 2025 Bai AD et al. *JAMA Network Open*.

eTable 7. Per-Protocol Analysis

	Patients No (%)		
	Oseltamivir treatment (N=7,468)	Supportive care (N=2,564)	Risk difference in % (95% CI) P-value
Primary outcome			
Death in hospital	249 (3.3)	108 (4.2)	Unadjusted: -0.9 (-1.8 to 0) P=0.04<br>Adjusted: -1.5 (-2.5 to -0.5) P=0.003
Secondary outcomes			
ICU transfer after 48 hours	88 (1.2%)	38 (1.5%)	Unadjusted: -0.3 (-0.9 to 0.2) P=0.28<br>Adjusted: -0.1 (-0.7 to 0.5) P=0.79
Readmission within 30 days	619 (8.3)	233 (9.1)	Unadjusted: -0.8 (-2.1 to 0.4) P=0.21<br>Adjusted: -0.8 (-2.3 to 0.6) P=0.26

CI = confidence interval; ICU = intensive care unit;

© 2025 Bai AD et al. *JAMA Network Open*.

eTable 8. Outcomes After Adjustment by Overlap Weighting of Propensity Scores That Includes mLAPS Score Within 24 Hours

	Risk difference in % (95% CI) P-value
Primary outcome	
Death in hospital	-1.8% (-2.8% to -0.9%) P<0.001
Secondary outcomes	
ICU transfer after 48 hours	-0.5% (-1.0% to 0.1%) P=0.12
Readmission within 30 days	-1.6% (-2.9% to -0.2%) P=0.02

CI = confidence interval; ICU = intensive care unit;

Notes on methodology: In a sensitivity analysis, mLAPS score was added to the covariates when estimating the propensity score. Complete case analysis was done, so patients with missing mLAPS score were excluded from this sensitivity analysis. Then overlap weighting and analysis of the primary and secondary outcomes were done.

© 2025 Bai AD et al. *JAMA Network Open*.
