# Supplementary material for: Oseltamivir Treatment vs Supportive Care for Seasonal Influenza Requiring Hospitalization
Source: JAMA Netw Open. 2025 Jun 10;8(6):e2514508. doi: 10.1001/jamanetworkopen.2025.14508 (PMC12152703; doi:10.1001/jamanetworkopen.2025.14508)
Supplement: Supplement 2. — Data Sharing Statement [file jamanetwopen-e2514508-s002.pdf]

Data Sharing Statement

Bai. Oseltamivir Treatment vs Supportive Care for Seasonal Influenza Requiring Hospitalization. *JAMA Netw Open*. Published June 10, 2025.
doi:10.1001/jamanetworkopen.2025.14508

Data
Data available: No
